# The efficacy of nursing intervention to reduce preoperative anxiety in patients with total knee arthroplasty: A protocol of prospective randomized trial

**DOI:** 10.1097/MD.0000000000022213

**Published:** 2020-09-18

**Authors:** Su Fu, Qin Wang, Chaofeng Fan, Yan Jiang

**Affiliations:** a Department of Neurological Comprehensive Ward; b Department of Orthopedics; c Department of Nursing, West China Hospital of Sichuan University/West China Nursing College, Sichuan, China.

**Keywords:** anxiety, motivational interview, nursing intervention, protocol, total knee arthroplasty

## Abstract

**Background:**

Some patients undergoing the total knee arthroplasty (TKA) have suboptimal postoperative results, and preoperative anxiety may be one of the reasons for these unsatisfactory results. We perform this randomized control study protocol to determine the effectiveness of nursing intervention, on the basis of motivational interview, to decrease the preoperative anxiety in patients receiving TKA.

**Methods:**

This is a double-blinded, single-center, placebo-controlled and randomized trial, which will be conducted from December 2020 to June 2021. The protocol of this study was approved by the West China Hospital of Sichuan University (W20200803-28). Sixty patients who will undergo TKA are included in our study. Patients are randomly divided into experiential group (with 30 patients) and the control group (with 30 patients). The control group and experimental group receive an informative and separate session via nursing about the operation preparation and operation process. Both the control group and the experimental group are given habitual treatment, but the experimental group need to receive additional motivational interviews. The primary outcomes are the Hospital Anxiety and Depression Scale and the Amsterdam Preoperative Anxiety and Information Scale. Secondary outcome is postoperative pain, which is assessed by visual analogue scale .

**Results:**

Figure 1 will display the comparison of preoperative and postoperative total average anxiety scores in control group and the experimental group.

**Conclusion:**

Preoperative psychological distress is familiar in our patients. We hypothesized that nursing intervention may be associated with reduced preoperative anxiety in the patients receiving TKA.

## 1 Introduction

Total knee arthroplasty (TKA) is quite successful surgery for many end-stage knee diseases in terms of functional recovery and pain relief.^[1,2]^ And the demand for primary total knee replacement is projected to grow by 3.5 million procedures in the United States alone by 2030.^[3]^ Although TKA is successful, approximately 20% of the patients were not satisfied.^[4,5]^ Previous researches indicated that an estimated 60% of patients report severe pain following TKA,^[6,7]^ which is the common reasons for dissatisfaction. However, the factors influencing the prognosis after TKA are both multifactorial and complex. About 30% of TKA patients have psychological distress before surgery.^[8]^ Anxiety disorders are considered common mental illnesses, with high worldwide prevalence.^[9]^ It has been reported that the patients who are considered anxious have worse outcome score than the patients with higher scores of psychosocial component. Even if we consider their importance, only a few researches have tried to determine the existence of psychological disorders such as anxiety. Pan et al^[8]^ have reported a steady increase in the prevalence of anxiety in patients with TKA each year. The anxiety was closely related to the efficacy of TKA.

The progress of nursing profession in nursing surgical service personnel is obvious; there are clear examples in safety policy of surgical patients, good instrument operation procedures and disinfection in operating room, nursing intervention is conducive to the safety of patients and reduced the risks related to surgery and anesthesia.^[10,11]^ Nevertheless, few study has reported the nursing intervention is beneficial to postoperative rehabilitation. Therefore, we perform this randomized control study protocol to determine the effectiveness of nursing intervention, on the basis of motivational interview, to decrease the preoperative anxiety in patients receiving TKA.

## 2 Methods

This is a double-blinded, single-center, placebo-controlled, and randomized trial that will be conducted from December 2020 to June 2021. An initial power calculation with 80% power and 5% significance indicated that a sample size of 60 patients in each group would be required. The protocol of this study was approved by the West China Hospital of Sichuan University (W20200803-28) and then is registered in research registry (researchregistry5908). It is performed in accordance with the SPIRIT Checklist for randomized studies.

### 2.1 Trial design and participants

Sixty patients who will undergo TKA are included in our study. In the random envelope, all participants will be assigned a random number via utilizing the random number table, and the result of allocation is hidden. Patients are randomly divided into experiential group (with 30 patients) and the control group (with 30 patients). Inclusion criteria contain people between the ages of 55 and 70; patients who underwent TKA procedure in our hospital within 2 months after the operation; the acceptance of patients to participate in this work. The exclusion criteria contains: people with the intellectual and cognitive impairment (behavioral-cognitive intervention); BMI above 35 kg/m^2^; the history of renal and hepatic dysfunction; and patients refused to participate in this study.

### 2.2 Intervention

To conduct the intervention, the participants from the control group and experimental group receive an informative and separate session via nursing about the operation preparation and operation process. At the first visit, a well-thought-out questionnaire is utilized to determine the general characteristics of the patient and to verify that the patient meet the criteria. Both the control group and the experimental group are given habitual treatment, but the experimental group need to receive additional motivational interviews. Before conducting the trial, both the groups were utilized the Amsterdam Preoperative Anxiety and Information Scale (APAIS),^[12]^ which is on the basis of 6-item questionnaire, and the response options were assessed from 1 to 5 by the Likert-type scale; 1 indicates not at all and 5 indicates extremely. The first 2 are associated with anxiety caused by anesthesia, and the numbers 4 and 5 are associated with the anxiety caused by surgery, and sum is regarded as the preoperative anxiety that ranging from 5 to 30. Hospital Anxiety and Depression Scale (HADS) ^[13]^ has been widely proved and then published in various medical fields, thus, it is a proper tool to evaluate the psychological states. HADS was primitively developed and proved via Snaith and Zigmond in 1983 with intention for the detection of anxiety and depression in adults between the ages of 16 and 65. It includes a subscale of depression (HADS-D) and anxiety (HADS-A). Each subscale consists of 7 items and is scored on the Likert 4-point scale (0–3), thus, each subscale can get a maximum of 21 points. The purpose of the questionnaire is to evaluate the status of participants in the past 2 weeks. In the investigations of adult population, the HADS displays good case-finding characteristic, diagnostic quality, and internal consistency.

The APAIS and HADS is applied by 2 nursing professionals, as evaluators, at the start and end of the procedure. Three motivational interview sessions are carried out over a period of 20 days, followed by a follow-up of 8 weeks. The sessions of motivational interview are chiefly on the basis of participants setting their goals and change their lifestyles slowly. Each session lasted about 40 minutes, in the 8 days before interview, their anxiety level and the trigger factors are investigated. The follow-up period is 2 months.

### 2.3 Outcome measures

The primary outcomes are the APAIS and HADS. Secondary outcome is postoperative pain, which is assessed by visual analogue scale.

### 2.4 Statistical analysis

All data analyses are implemented through utilizing SPSS for Windows Version 13.0. All the data are represented with proper characteristics as median, mean, percentage, as well as standard deviation. Mann-Whitney *U* test or the independent samples *t* test was utilized to compare the 2 groups. *χ*^2^ detection is utilized to compare the categorical variables among the groups. The analysis of repeated measurement of the variance is applied to analyze the repeated data. A *P* < .05 is regarded the significant in statistics.

## 3 Results

**Figure 1:**
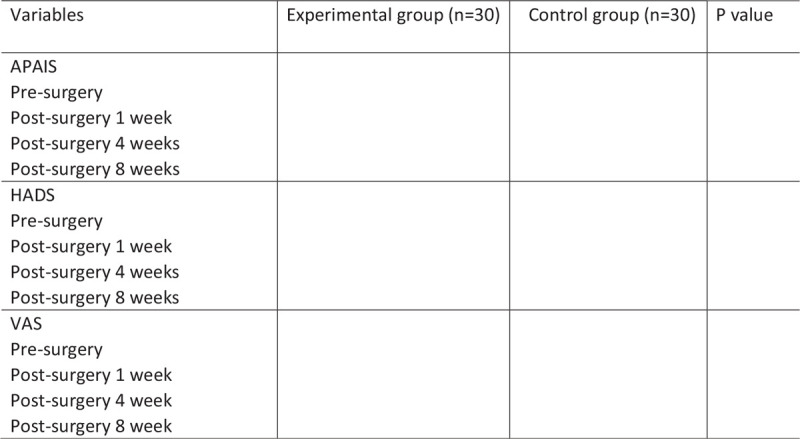
Comparison of the total average score of preoperative anxiety in the study groups before and after the procedure.

Figure 1 will display the comparison of preoperative and postoperative total average anxiety scores in control group and the experimental group.

## 4 Discussion

This is the first randomized controlled trial to assess the effectiveness of motivational interview-based nursing intervention in decreasing the preoperative anxiety in the patients receiving TKA for a period of 2 months. Despite advances in the nursing interventions, anxiety is still a problem for patients. Sadati et al^[14]^ reported that the nursing visits before operation could reduce the preoperative anxiety level and decrease the postoperative complications of laparoscopic cholecystectomy candidates. Anxiety is a kind of temporary emotional state of fear, nervousness, tension, and highly active autonomic nervous system.^[15]^ The anxiety-related events can affect rehabilitation significantly, including the admission and hospital environment, anesthesia, and surgery. Anxiety can increase pain after operation, increase the consumption of analgesics, and prolong the length of hospital stay, which directly affects the medical expenses.^[16,17]^ Nursing intervention is considered to be a good way to control these worsening effects. A growing body of studies have recognized the importance of communication skills.^[18,19]^ The nurses must be exposed to and care for the patient after surgical event, which can be assessed and followed up via conducting the motivational interviews.^[20]^ It is regarded as an effective method to improve personal behavior and attitude through trust and persuasion.

## 5 Conclusion

Preoperative psychological distress is familiar in our patients. We hypothesized that nursing intervention may be associated with reduced preoperative anxiety in the patients receiving TKA.

## Author contributions

Su Fu writes the manuscript; Qin Wang collects and analyzes data; Chaofeng Fan edits the manuscript; Yan Jiang designs the manuscript. All authors approve the submission.

**Formal analysis:** Qin Wang.

**Investigation:** Qin Wang.

**Methodology:** Qin Wang.

**Writing – original draft:** Su Fu.

**Writing – review & editing:** Chaofeng Fan.

## Abbreviations

Abbreviations: APAIS= Amsterdam Preoperative Anxiety and Information Scale, HADS= Hospital Anxiety and Depression Scale, TKA= total knee arthroplasty.
